# Nutraceutical Value of Finger Millet [*Eleusine coracana* (L.) Gaertn.], and Their Improvement Using Omics Approaches

**DOI:** 10.3389/fpls.2016.00934

**Published:** 2016-06-29

**Authors:** Anil Kumar, Mamta Metwal, Sanveen Kaur, Atul K. Gupta, Swati Puranik, Sadhna Singh, Manoj Singh, Supriya Gupta, B. K. Babu, Salej Sood, Rattan Yadav

**Affiliations:** ^1^Department of Molecular Biology and Genetic Engineering, G.B. Pant University of Agriculture and TechnologyPantnagar, India; ^2^Institute of Biological, Environmental and Rural Sciences, Aberystwyth UniversityAberystwyth, UK; ^3^ICAR–Vivekananda Institute of Hill AgricultureAlmora, India

**Keywords:** finger millet, nutraceuticals, functional food, health benefit, nutritional security, nutrition

## Abstract

The science of nutritional biology has progressed extensively over the last decade to develop food-based nutraceuticals as a form of highly personalized medicine or therapeutic agent. Finger millet [*Eleusine coracana* (L.) Gaertn.] is a crop with potentially tremendous but under-explored source of nutraceutical properties as compared to other regularly consumed cereals. In the era of growing divide and drawback of nutritional security, these characteristics must be harnessed to develop finger millet as a novel functional food. In addition, introgression of these traits into other staple crops can improve the well-being of the general population on a global scale. The objective of this review is to emphasize the importance of biofortification of finger millet in context of universal health and nutritional crisis. We have specifically highlighted the role that recent biotechnological advancements have to offer for enrichment of its nutritional value and how these developments can commission to the field of nutritional biology by opening new avenues for future research.

## Introduction

The World Health Organization (WHO) recognizes that maintenance of optimum global public health requires not only prevention of early onset of chronic diseases but also promotion of a healthier aging and ultimately prolongation of life. Despite its tremendous advancement, medical science still faces a dual challenge to promote public health: side effects of medicines and their economic encumbrance. Going back to a well-recognized dogma of ancient Indian Siddha literature and Hippocrates ‘food is medicine and medicine is food,’ diet and dietary habits have been established to play important role for exalted health benefits based on different properties of foods (Dev et al., 2011). Thus, growing demand for healthier food products is stimulating innovation and development at the international scale. This research progress has advanced the emerging group of health food termed as nutraceuticals with an estimated global market of around 117 billion US Dollars (Chauhan et al., 2013). The word ‘nutraceutical’ was coined by Stephen DeFelice in 1989 and refers to a food or food product sold as pills, powders, or other medicinal forms to provide potential health benefits (Keservani et al., 2010; Dev et al., 2011). Existing health reports suggest several physiological benefits of nutraceuticals in prevention, protection or treatment against several chronic ailments. This relationship can be visualized in day-to-day life, for example importance of calcium in preventing osteoporosis (Srivastava and Deal, 2002; Lanham-New, 2008), folate in the prevention of infant neural tube defects (Lumley et al., 2001; Czeizel et al., 2013) and the role of decreasing dietary fat and increasing fiber from grains in the prevention of colon cancer (Schatzkin et al., 2007; Lattimer and Haub, 2010) etc. Thus, any significant alteration of nutrients due to improper dietary practices affects the health outcome. This area provides an opportunity for discovery and development of specific food components which can act as candidate agents for developing nutraceutical compounds. Given their health benefitting values, whole-grain cereals can be acclaimed as major nutraceutical candidates for human consumption. This review not only presents a balanced view from the perspective of biofortification of one such crop, finger millet [*Eleusine coracana* (L.) Gaertn.], possessing wholesome traits to meet global nutritional infirmity, but also explores its nascent significance as means of advancing the future of nutraceutical industry.

## Nutrigenetics and Nutrigenomics Approach To Study Nutrient-Gene Interaction

The health and nutrition-related disorders in various populations have been correlated to the differential interaction of nutrient-dependent genetic diversity (Stover, 2006). Research in the post-genomic era has revealed that external factors like diet can influence intrinsic physiology through molecular modulation of genes. Capitalizing on this progress, new approaches that analyze effect of nutritional environment on genes or of genetic architecture on nutrient metabolism have emerged in the last decade. Termed as nutrigenomics and nutrigenetics, these approaches explore the intricate association of nutritional molecules and genetic polymorphisms within the biological system in a holistic manner to optimize health through the diet (Mutch et al., 2005).

The scientific area of ‘nutrigenomics’ is actually integration of genomics into nutrition sciences. It uses functional genomic tools for genome-wide identification of genes which are influenced by food components, resulting in alterations in homeostatic pathways in a biological system. It is a challenging area as a single nutrient can have different targets, affinities or specificities based on population under study. Quantitative techniques for integrated or independent study of nutrients on subtle changes in gene expression (transcriptomics), proteins (proteomics), or metabolites (metabolomics) in single cell as well as whole organism have increased the knowledge about nutrient-gene interaction aiding nutritional research tremendously. With the completion of several genome-sequencing projects, new genes involved in several metabolic pathways have been identified. Microarray analyses in nutrigenomic studies have defined gene expression pattern changes for 1000s of such genes (Blackburn et al., 2006). Dietary components can modify translation and post-translational events changing the protein structure or function (Trujillo et al., 2006). Proteomic study can provide more dynamic information against cellular responses toward a particular nutritional challenge, however, not a sizeable percentage of nutriproteomics-related publications are reported, creating a knowledge gap. The most routinely performed experiments in nutritional biology comprise the area of metabolomics involving large-scale analyses of multiple metabolites in relation to nutrient exposure and their bioavailability. Complete profiling of individual metabolites, for example cholesterol, triglycerides, thiamin, riboflavin, can be generated by analyzing biofluids. Although metabolites are not a direct gene product, their formation and role depends on the multiple genes and can determine regulation of function. It is still not possible to perform a whole organism proteome or metabolome analysis, but these can identify nutritional biomarkers which can distinguish between individuals based on their dietary intake, nutritional status, metabolism and its functional effects on disorder (Combs et al., 2013). Further, high throughput screening systems have enabled the discovery of new functional compounds, metabolic pathways and responsible genes from cell lines. Despite this, for precise recognition of a nutraceutical, it is imperative to understand the reaction of these metabolic pathways, in general and specific gene, in particular, toward nutrient and non-nutrient dietary components. Thus, through nutrigenomics and incorporating data mining bioinformatics tools, one can identify the lead nutraceutical molecule with potential for preclusion and therapy of nutrition-related disorders.

Individual preferences and differences in diet and lifestyle also affect the genetic makeup and such food-responsive gene-based variation can be understood by nutrigenetics. Conversely, it also aims to study that how genic variations such as single nucleotide polymorphisms coordinate individualistic responses to diet in the whole population. It is driven by the hypothesis of selection of genes based on their ‘suspected’ effect on health, for example, insulin signaling genes for diabetes or lipoprotein genes as candidates for obesity. Nutrigenetics can reveal polymorphism in response to food components in genetically diverse population and identify groups which could be predisposed to nutrition-relation disorders or those which could gain substantial benefit from dietary intervention. Thus, nutrigenomics and nutrigenetics research needs to be coupled with a number of other academic fields like molecular biology, molecular nutrition, pharmacogenomics, and molecular medicine for a customized designing of knowledge-based functional foods on a large scale.

In short, these gene-diet interaction studies establish the impact of macro- and micro-nutrients on general well-being of humans. Many of these nutrients are essential to the body, that is, they require external intervention from other sources. Plant-derived foods are richest and most economically feasible resource of nutrition to a global population in both developing and developed economies. Among them, millets are one of the oldest and possibly the first cereal grain to be used for domestic purposes by humans. At present, they are ranked among top 10 most important grains sustaining about one third of the world’s population (Changmei and Dorothy, 2014). Four different types of major millets are pearl millet [(*Pennisetum glaucum* (L.) R. Br.], finger millet [*E. coracana* (L.) Gaertn.], foxtail millet [*Setaria italica* (L.) P. Beauvois.], and proso millet [*Panicum miliaceum* (L.)]. They are considered as a storehouse of nutritional properties and remedy for malnutrition that affects a vast majority of populations in developing countries (Millet Network of India, 2010). It is acclimatized to a broad range of agro-geographic conditions including vast dry land areas. Thus, their vertical integration into agriculture and functional food manufacturing can complement traditional crops and positively impact economic and nutraceutical development. This review focuses on the importance of finger millet for nutraceutical research. Further, we also discuss the role that biotechnology and nutrition biology have to play in order to utilize this crop for developing functional food for human consumption.

## Finger Millet: Wonder Grain Of Nutritional Importance

Finger millet belongs to the family Poaceae and is more commonly known as ragi or madua in India, rapoko in South Africa and dagusa in Ethiopia (Obilana and Manyasa, 2002; Ignacimuthu and Ceasar, 2012)^1^. Globally, 12% of the total millet area is under finger millet cultivation, covering more than 25 countries of Africa and Asia^2^. It forms a predominant staple food for people living on marginal lands and with limited economic resources. An agronomically sustainable crop, it can grow on marginal lands, high altitudes and can easily withstand drought and saline conditions, requires little irrigation and other inputs and yet maintain optimum yields.

Finger millet has been perceived as a potential “super cereal” by the United States National Academies being one of the most nutritious among all major cereals (National Research Council, 1996; **Table 1**). From the nutritional perspective, finger millet is considerably rich in minerals and its micronutrient density is higher than that of the world’s major cereal grains; rice and wheat (Antony and Chandra, 1998; Vadivoo et al., 1998). Specifically, it is the richest source of Calcium among cereals with up to 10-fold higher Calcium content than brown rice, wheat or maize and three times than that of milk. It is also rich in iron and fiber, making this crop more nutritive as compared to other most commonly used cereals. Finger millet is enriched in the essential amino acids like lysine (McDonough et al., 2000) and methionine which are important in human health and growth but remain absent from most other plant foods. In addition, it also contains useful amounts of the two polyunsaturated fatty acids- linoleic acid and α-linolenic acid (Fernandez et al., 2003), metabolized products of which facilitate normal development of the central nervous system (Birch et al., 2007; Jacobson et al., 2008). It also contains both water soluble and liposoluble vitamins: thiamin, riboflavin, niacin, and tocopherols (Obilana and Manyasa, 2002). As finger millet is often grown and consumed primarily in developing countries by small-hold farmers with limited agronomic resources, it is often referred to as a “crop for the poor” or a “famine food” (National Research Council, 1996). Despite the well-documented health benefits of finger millet, only a limited progress has been made in context to its nutraceutical applications as functional food. Although, it may have remained underutilized by the general population due to either unawareness or apprehension, its immense potential for enhancing the nutritional and therapeutic attributes by acting as functional food cannot be ignored.

**Table 1:** Nutritional composition of main millets in comparison to major cereals (@ 12% moisture; per 100 g edible portion).

Nutrients	Finger millet^1,2^	Pearl millet^1,2^	Foxtail millet^1,2^	Proso millet^1,2^	Wheat^1,2^	Rice (white, milled, raw)^1,2^	Rice (brown, medium grain, raw)^3^	Corn grain (white)^3^	Sorghum^3^	Oats^3^	Barley (pearled, raw)^3^
**Proximate composition**
Moisture (g)	13.1	12.4	11.2	11.9	12.8	13.7	12.4	10.4	12.4	8.2	10.1
Energy (kcal)	336	361	331	341	346	345	362	365	329	389	352
Protein (g)	7.7	11.6	12.3	12.5	11.8	6.8	7.5	9.4	10.6	16.9	9.9
Fat (g)	1.5	5	4.3	1.1	1.5	0.5	2.7	4.7	3.5	6.9	1.2
Total dietary fiber (g)	11.5	11.3	2.4	–	12.5	4.1	3.4	7.3	6.7	10.6	15.6
Carbohydrate (g)	72.6	67.5	60.9	70.4	71.2	78.2	76.2	74.3	72.1	66.3	77.7
Minerals (g)	2.7	2.3	3.3	1.9	1.5	0.6	–	–	1.6	–	–
**Minerals and trace elements**
Calcium (mg)	350	42	31	14	30	10	33	7	13	54	29
Iron (mg)	3.9	8	2.8	0.8	3.5	0.7	1.8	2.7	3.36	4.7	2.5
Magnesium (mg)	137	137	81	153	138	64	143	127	165	177	79
Phosphorus (mg)	283	296	290	206	298	160	264	210	222	523	221
Manganese (mg)	5.94	1.15	0.6	0.6	2.29	0.51	–	–	0.78	–	–
Molybdenum (mg)	0.102	0.069	0.7	–	0.051	0.05	–	–	0.039	–	–
Zinc (mg)	2.3	3.1	2.4	1.4	2.7	1.3	2.02	2.21	1.7	3.97	2.1
Sodium (mg)	11	10.9	4.6	8.2	17.1	–	4	35	2	2	9
Potassium (mg)	408	307	250	113	284	–	268	287	363	429	280
**Vitamins**
Thiamine (mg)	0.42	0.33	0.59	0.2	0.45	0.06	0.41	0.39	0.33	0.76	0.19
Riboflavin (mg)	0.19	0.25	0.11	0.18	0.17	0.06	0.04	0.2	0.096	0.14	0.11
Niacin (mg)	1.1	2.3	3.2	2.3	5.5	1.9	4.3	3.6	3.7	0.96	4.6
Total Folic acid (μg)	18.3	45.5	15	–	36.6	8	20	–	20	56	23
Vitamin E (mg)	22	–	–	–	–	–	–	–	0.5	–	0.02

*(1) Gopalan et al. (1999).*

*(2) Gopalan et al. (2004).*

*(3) USDA National Nutrient Database for Standard Reference, Release 28 (2016).*

## Processing and Utilization

Finger millet grains can be processed in several ways depending upon the ultimate utilization. In order to develop consumable products, the different processing techniques include milling, malting, popping, puffing, flaking, debraning, etc. (Shobana et al., 2013). They are crushed in a roller mill, like wheat, and win-mowed, to give coarse flour which is utilized as porridge. The whole grain or refined flour is used for preparations like cakes, pudding, porridges and also local Indian cuisines like bhakri, dhebra, papad, etc. Besides having a good shelf life, the flour is fat and gluten-free, is easily digestible and requires little cooking (Saturni et al., 2010). The malted grain’s flour is also good nourishing food for infants and invalids. In several regions, finger millet grains are malted and fermented to obtain traditional beer. Presently, it is consumed as a staple food only by a small section but its appealing nutritional traits have now created wide interest among the scientists to explore the nutraceutical properties of finger millet.

## Exploring Properties of Finger Millet For Nutraceutical Development

Recent research has shown that diets rich in plant-based foods, particularly whole-grain cereals, are protective against several degenerative diseases such as diabetes, cardiovascular diseases, few types of cancers, metabolic syndrome and Parkinson’s disease (Manach et al., 2005; Scalbert et al., 2005; Fardet et al., 2008). In spite of the dearth of literary evidence on role of finger millet for disease prevention, its relation to alleviate health benefits is quite substantial. It is a storehouse of salubrious properties being rich in proteins, dietary fibers, minor and major nutrients, and phytochemicals required for human health (**Table 2**). However, these ingredients need to be isolated and developed as nutraceuticals and functional food, without disturbing the inherent functional and nutritional traits and in a cost-efficient manner. Nutraceutical properties of finger millet are discussed in details in the following sections.

**Table 2:** Nutraceutical properties of finger millet.

S. no.	Nutrients	Ingredients	Functional properties	Nutraceutical properties	Reference
1	Phytochemicals	Tannins, steroids, polyphenols, alkaloids, terpenoids, cardiac glycosides and balsams, lignans, phytooestrogens, phytocyanins.	Antioxidants, inhibitors of biological oxidation, antifungal activity, immune modulators, detoxifying agents, protect against age-related degenerative diseases.	Cardiovascular diseases, Cognition and neuro degenerative diseases, cancer and aging, blood glucose and cholesterol lowering, nephro protective and anti-cataractogenic properties, anti-diabetic.	Rao and Muralikrishna, 2002; Hegde et al., 2005; Rao et al., 2011; Mathanghi and Sudha, 2012; Saleh et al., 2013
2	Proteins	Prolamins, lobulins, albumins, CBPs.	High proportion of essential amino acids and bioactive peptides.	Protein energy malnutrition and maintaining homeostasis in various ailments, natural relaxant.	Indira and Naik, 1971; Ravindran, 1991; Antony et al., 1996; Mathanghi and Sudha, 2012
3	Glycoproteins and lower fat contents	Biologically important glycosyl moieties	Reduction of glycosylation	Aging, extremely good shelf life	Antony et al., 1996; Mathanghi and Sudha, 2012
4	Carbohydrates	Free sugars, starch, cellulose, pentosans, xylose, fructose, glucose, sucrose, maltose, raffinose, maltotriose, higher oligosaccharides, resistant starch, hemicellulose	Slow and gradual digestibility	Anti- diabetic and obesity	Mathanghi and Sudha, 2012
5	Fibers	Soluble fiber	Lowering of plasma glucose and cholesterol, weight loss due to low calorie intake, laxative, good bowel movement, and reduction in blood cholesterol and sugar	Anti-diabetic, cardiovascular diseases, gastro-intestinal disorders, avoid gallstones, constipation, cancer, and aging	Mathanghi and Sudha, 2012; Saleh et al., 2013
6	Vitamins	Water soluble, fat soluble, mostly thiamine, riboflavin, niacin	Aid absorption of iron	Anemia	Mahudeshwaran and Ayyamperuman, 1970; Obilana and Manyasa, 2002; Gopalan et al., 2004; Tatala et al., 2007
7	Minerals	Potassium, sodium, calcium, magnesium, zinc, manganese, iron, lead	High mineral content in seeds	Osteoporosis, anemia	Antony and Chandra, 1998; Obilana and Manyasa, 2002; VanderJagt et al., 2007; Platel et al., 2010

### Anti-oxidant and Anti-aging Properties

Chronic diseases like diabetes, heart disease, cognition disease, cancer and several other normal functions in humans have been linked to oxidation of cellular molecules by reactive oxygen and nitrogen species. Several phytochemicals act as dietary antioxidants to defend against the oxidative damage and maintain a proper physiological balance. In the past few years, dietary plant polyphenols have received tremendous attention from health professionals, nutrition scientists, and consumers for their pleotropic health benefits like reduced risk of cancer, cardiovascular and neuro-degenerative diseases, infections, aging and diabetes (Kaur and Kapoor, 2001; Scalbert et al., 2005; Tsao, 2010). Finger millet grains, particularly the seed coat, contain high amount of various phenolic compounds (mostly derivatives of benzoic acid) which have been reported to exhibit antioxidant activity (Rao and Muralikrishna, 2002; Hegde et al., 2005; Chandrasekara and Shahidi, 2010). On an average, white finger millet contains 0.04–0.09% polyphenols and brown varieties have 0.08–3.47% (Chethan and Malleshi, 2007). Rao and Muralikrishna (2002) found proto-catechuic acid (45.0 mg/100 g) as the major free phenolic acid in finger millet grains. In another study of total phenolic compounds in finger millet, it was found to have 85% benzoic acid derivatives (gallic acid, protocatechuic acid, *p*-hydroxybenzoic acid, vanillic acid, syringic acid) while rest of the fraction were either cinnamic acid derivatives (ferulic acid, *trans*-cinnamic acid, *p*-coumaric acid, caffeic acid, sinapic acid) or flavonoids (quercetin, Proanthocyanidins like condensed tannins; Chethan and Malleshi, 2007). Among bound phenloic acids, ferulic and *p*-coumaric acid are the major fractions and account for 64–96 and 50–99% of total ferulic and *p*-coumaric acid content of finger millet grains, respectively, (Devi et al., 2014). A diet with 55% finger millet increased the activities of antioxidant enzymes like catalase, glutathione peroxidase, and glutathione reductase in rats pointing to its protective role (Hegde et al., 2005). Finger millet can also inhibit collagen cross-linking (Hegde et al., 2002), thereby can be used to slow down aging by reducing the stiffness of elastic tissues in tendons, skin and blood vessels. However, processing of finger millet like thermal or hydrothermal treatments, germination, decortication, or fermentation has been found to generally decrease the polyphenol levels resulting in a reduced radical quenching ability than that of the unprocessed grain (Sripriya et al., 1996; Towo et al., 2003; Rao and Muralikrishna, 2007; Shobana and Malleshi, 2007). Thus, more experiments should be performed to confirm the best applicability before this natural antioxidant cereal can be potentially developed as a nutraceutical and functional food ingredient.

### Anti-carcinogenic Properties

In recent years, prevention of diseases in natural ways has gained colossal interest. Protection against cancer by the switching to healthier food alternatives is very attractive, especially when taking into consideration the limited progress achieved by traditional medicine. The frequency or rate of spontaneous or induced tumors can be reduced by incorporation of anti-carcinogenic food in the diet. Phytochemicals and antioxidants are two particular nutraceutical components that have extensive anti-carcinogenic properties by acting as terminators of free radical and singlet oxygen species (Shahidi et al., 1992). As finger millet has a variety of such compounds they may suppress excessive cellular oxidation providing protection from different types of cancers prevalent in human population. Ferulic acid has been found to have blocking effect on induced carcinogenesis in tongue and colon of rats (Mori et al., 1999; Kawabata et al., 2000), and in breast cancer cells (Choi and Park, 2015) implicating that this major constituent of bound phenolic acids in finger millet, a may act as a natural bioactive chemotherapeutic agent against cancer. Additionally, being rich in nitriloside (Vitamin B17; a molecule that is specifically poisonous to only the cancer cell), finger millet grains can prove an efficient source in curing different cancers without any harmful side-effect (Griffin, 1974). It has been reported that incidences of oesophageal cancer are lowered by consumption of sorghum and millet as compared to wheat or maize (van Rensburg, 1981). Cancer initiation and progression in several tissues may be reduced due to millet’s phenolic components, tannins and phytate (Graf and Eaton, 1990; Chandrasekara and Shahidi, 2011a,b). Singh et al. (2015) reported the anti-cancer effects of finger and pearl millet phenolics through cyto-toxicity assay on HepG2 hepatic cancer cell lines and in silico studies. Several case-control and cohort studies have established the role of whole cereal-based dietary fiber against breast cancer (Challier et al., 1998; Cade et al., 2007). Dietary fiber from finger millet may also have similar effects; however, there is a need to prove their role in suppressing the cancerous growth of different neoplasms.

### Anti-diabetogenic Properties

Diabetes mellitus or diabetes is a major health concern on rapid rise in India as well in many other nations. It is a chronic metabolic disorder diagnosed by hyperglycemia either due inadequate insulin production (type-1) or because of mutual endurance to insulin secretion and action (type-2). Clinically, -prandial hyperglycemia can be managed by chemical synthetic inhibitors of α-glucosidase and pancreatic amylase, the potential safety of natural inhibitors is overt. Phenolic extracts from finger millet seed coats were shown to be effective inhibitors of these enzymes (Shobana et al., 2009). This property is greatly beneficial to deter and manage the epidemics of diabetes because of the lowered blood glucose levels (Shobana et al., 2009; American Diabetes Association, 2010; Kim et al., 2011). Finger millet based food formulations and preparations show lower glycemic index and induce lower glycemic response (Shobana et al., 2007; Shukla and Srivastava, 2014). Intake of dietary calcium and magnesium was suggested to reduce the type-2 diabetes risk in two independent studies (Pittas et al., 2006; van Dam et al., 2006). Thus, the abundance of these minerals in finger millet (**Table 1**) could be indirectly connected with its ability to alleviate type-2 diabetes risk. It has been reported that consumption of multigrain flour containing 30% finger millet proportion significantly lowered the plasma glucose levels (Pradhan et al., 2010), which could be attributed to the dietary fiber-mediated delayed carbohydrate digestibility. The presence of certain anti-nutritional factors in whole finger millet fractions (like tannins, phenolics, and phytates) may also help to lower the glycemic response due to decreased starch digestibility and absorption (Kumari and Sumathi, 2002). The antioxidant properties of whole grain finger millet meals were shown to positively affect the glycemic status in alloxan-induced hyperglycemic rats (Hegde et al., 2005). Additional health concern for diabetic people is delay in wound healing and cataract. Independent experiments on rat models have successfully proved than finger millet diet hastened the dermal wound healing and delayed the onset of cataractogenesis (Rajasekaran et al., 2004; Shobana et al., 2010). Very small amount of finger millet methanolic extracts (3 mg) were found to significantly inhibit glycation of collagen *in vitro* as compared to similar effects provided by 125 mg of a chemical antiglycating agent, implicating health benefit in pathogenesis of diabetes mellitus complications (Hegde et al., 2002). Therefore, finger millet may be considered as a functional ingredient for management of diabetes and associated complications.

### Cardio-Protective and Anti-hyperlipidemic Properties

Disorders of the heart and blood vessels or cardiovascular diseases have emanated as the primary cause of mortality worldwide. The problem is aggravated due to risk factors like abnormal blood pressure, elevated cholesterol, hypertension or depression, obesity and diabetes. Incidence of cardiovascular diseases is less in millet consumers than communities that do not consume millet (Gopalan, 1981). This may be because they act against hyperlipidemia by reducing triglycerides and total cholesterol in the serum of hyperlipidemic rats (Lee et al., 2010). Thus, a finger millet constituent diet leads to lower lipid peroxidation which pares down arteriosclerosis and thus provide valuable protection against stokes or heart attack. This is also supported by a recent study where a multigrain formulated diet with finger millet as one of the constituent, was able to effectively control the lipid and antioxidant metabolism in high cholesterol intake rat models (Vasant et al., 2014). Phenolic extracts from different millets were found to inhibit oxidative modification of LDL cholesterol in an *in vitro* copper-mediated human LDL cholesterol oxidation system, however, this function was varietal or dose-dependent with respect to specific phenolics (Chandrasekara and Shahidi, 2012a). Soluble dietary fiber component of the grain reduces reabsorption of bile acids (which are biosynthesized from cholesterol) and decrease the LDL cholesterol (Chandrasekara and Shahidi, 2012b). Fermentation of finger millet is also an economical alternate source to harness important metabolites, like statin and sterol, which are commonly used in hypercholesteraemic therapy (Venkateswaran and Vijayalakshmi, 2010). Statin acts as an enzyme inhibitor of the cholesterol biosynthetic pathway whereas dietary sterol helps in reducing serum LDL (bad cholesterol) level without affecting HDL (good cholesterol; Law, 2000; Ostlund, 2002). Thus, germination and fermentation provides scope for development of value-added finger millet-based functional food.

### Prevention of Gastro-Intestinal Disorders and Malnutrition

Celiac disease is amongst the most common lifelong auto-immune genetic disorders globally (Catassi and Fasano, 2008). This enteropathic disease is triggered specifically by ingestion of a group of proteins, called gluten, found commonly in cereals like wheat, rye and barley. The only treatment on offer is coherence to a strict diet of gluten-free foods including rice, corn, sorghum, millet, amaranth, buckwheat, quinoa, wild rice, and oats (Thompson, 2009). Finger millet can act as a wheat substitute given their quite similar protein structure yet the non-glutinous nature. In this respect, they have considerable potential to be developed as functional food (Taylor et al., 2006; Taylor and Emmambux, 2008; Chandrasekara and Shahidi, 2012b).

Finger millet is also rich in a mixture of soluble and insoluble dietary fibers or roughage that are resistant to breakdown during digestion and help to prevent gastro-intestinal disorders, colon cancer, coronary heart disease, and diabetes (Anderson et al., 2009). Due to its high cellulose content, the insoluble fiber of finger millet helps to bulk the stool, acts as laxative to stimulate bowel mobility and prevents constipation by retaining water in feces and promoting peristalsis. The soluble fibers, on the other hand, assist lubrication and soothing of an inflamed digestive tract. In addition to the fibers, the polyphenols can also help to reduce peptic inflammation and exhibit anti-ulcerative characteristics (Chethan and Malleshi, 2007).

Today our world in challenged by the radical paradox of malnutrition: undernutrition (deficient intake of nutrients) and obesity (excessive dietary consumption). Being a highly balanced and easily digestible grain, finger millet presents an ideal diet for both the groups. For the people suffering from protein-energy malnutrition, it is an excellent source carbohydrate (80%) and protein (7–9%) with essential amino acids like valine, methionine, and tryptophan which otherwise are scarcely present in a vegetarian diet. Further, minerals like calcium, phosphorus, potassium, and iron and vitamins like and thiamine, niacin, and riboflavin are also abundant (**Table 1**). Finger millet is low in fat content and is good for obese people. Supplementation with finger millet bran helped to prevent high-fat diet-induced obesity and increased the abundance of beneficial gut microflora in rodent models (Murtaza et al., 2014). Additional advantage to this group of people is that the carbohydrate component is slowly digestible and takes longer time to get absorbed. Consumption of food products derived from its grain can increase the satiety index, reduce excessive calorie intake and thus can promote weight loss.

### Prevention of Osteoporosis and Other Bone Ailments

Osteoporosis is a “silent” ailment of bone mass loss. The WHO has proposed osteoporosis as the second major global healthcare concern after cardiovascular diseases (Haldipur, 2004). Consuming conventional calcium mineral supplements has some side effects (Straub, 2007) and may not be affordable to all sections of society. High dietary intake of naturally available calcium facilitates prevention of bone diseases like osteoporosis. Finger millet is a reasonably good source of calcium with up to 350 mg/100 g of Calcium present in the seeds, which is 5–10 times higher than other cereals (Panwar et al., 2010a; Sanwalka et al., 2011; Kumar et al., 2013). In comparison, cow’s milk, which is common source of Calcium for many people, contains on average 112 mg Calcium/100 g milk (Wijesinha-Bettoni and Burlingame, 2013). Unlike milk, however, the absence of lactose sugar makes it an easily digestible alternate source nutrient for lactose-sensitive patients and weaning babies. Therefore, products derived from finger millet can be utilized in bone mass development in growing children as well as for preventing osteoporosis and other bone ailments in adults and aging population. Thus, all the nutritional significance of finger millet must be properly translated to nutraceutical development and applied to other staple crops for their possible enrichment.

## Scientific Developments For Elucidating Nutrient Trait Pathways In Finger Millet

In order to harness nutritional benefit from finger millet and to develop it as a functional crop for biofortification, novel technologies which are capable of assessing a complete biological network must be employed. A detailed insight into the effects of genes, protein or metabolite profiles can provide a comprehensive understanding of nutrient trait pathways. However, there is there is dearth of information of specific gene or gene products involved in a metabolic pathway of interest in finger millet. Therefore, for elucidating the candidate genes involved in the nutrient pathways, mutagenesis or genome alteration strategies for producing a particular phenotype must be employed. Analogy can be driven by the example of ‘golden rice’ which has effectively deflected the vitamin A deficiency by genetic engineering of β-carotene biosynthesis pathway in rice endosperm (Ye et al., 2000). Genetic engineering or marker assisted selection can be used for introgression candidate gene of nutritional importance. Hence, research should be focused on the identification of candidate genes involved in different nutrient pathways. The subsequent section discusses how the tools and techniques in the areas of genomics, transcriptomics, proteomics, and metabolomics can to be applied to improve understanding and accelerate resources for nutritional research in finger millet.

### Genomics-Assisted Gene Discovery for Nutritional Trait Improvement

In the last several years, crop improvement has substantially gained from the continuous progress and coupling of research efforts in the areas of plant biotechnology, molecular breeding, and genomics. Advances in genomics imply that large-scale identification of genes or gene-products of an organism or whole populations is now possible through next-generation sequencing. This high-throughput platform further allows an efficient genome-wide analysis of genotypic variation and can thus be employed to understand genetic basis of nutritional traits in finger millet. Genome sequencing efforts in finger millet have been initiated in 2014, but are still in its nascent stage^3^. Mysore and Baird (1997) have estimated the genome size (2C) of tetraploid finger millet (2n = 4x = 36) to be in the range of 3.34–3.87 pg. In the absence of availability of a genomic sequence, breeding for improved nutritional content will still have to rely on traditional genotyping and mapping using molecular markers. On the other hand, mutagenesis-based high throughput functional genomics approaches like TILLING & ECOTILLING which are not dependent on genome sequence can be used for polyploid species like finger millet (Tadele et al., 2010).

### Utilization of Germplasm Resources in Finger Millet

The primary center for origin and domestication of cultivated finger millet (*E. coracana* subsp. *Coracana*) is predicted to be the uplands of East Africa, especially Ethiopia and Uganda around 5000 years ago from wild finger millet race (*E. coracana* subsp. *Africana*; The Global Crop Diversity Trust, 2012). *E. coracana* subsp. *Coracana* was forbearer of the Afro-asiatic lowland race by migration to the African lowlands followed by an eastward sea-trade movement to India around 3000 BCE generating a secondary set of germplasm diversity (Dida et al., 2008). Given the long history of domestication, finger millet germplasm now suffers from low levels of genetic diversity (Agrawal and Maheshwari, 2016). Prolonged depletion of genetic diversity in the present breeding programs necessitates maintenance of gene pool variation by conservation of rich germplasm sources and promoting its use internationally. Due to insufficient evaluation data, millet germplasm collections have remained underutilized, in comparison to other major cereals (Upadhyaya et al., 2006). Their use has been enhanced by developing sets of germplasm core collections which represent almost all the genetic diversity present in the crop and its wild relatives and can be used for mining of important alleles for traits of interest, like of nutrient quantity (Frankel and Brown, 1984). The International Crops Research Institute for the Semi-Arid Tropics (ICRISAT), Hyderabad conserves 5940 accessions of finger millet germplasm in their gene bank^4^. To utilize this large germplasm collection for breeding strategies, Upadhyaya et al. (2006) have developed a set of core collection incorporating about 10% (622 accessions) from the entire conserved assembly. These studies have paved the way toward genetic improvement of finger millet for higher yield and productivity. There are a few reports on the characterization of finger millet core collection genotypes based on their grain nutritional quality (Upadhyay et al., 2011). This study can assist in planning crosses to breed finger millet for enhanced grain nutrients and to develop multiple nutrient content-based mapping populations.

### Development of Molecular Markers for Genetic Diversity and Gene Discovery

A reliable knowledge of genetic diversity is important to select genetically distinct germplasm for hybridization programs to achieve better crops. Studying genetic and genomic polymorphism by the use of molecular markers has now become an indispensable tool. Although, researchers have developed several markers in finger millet, these studies are limited with respect to the small number of genotypes used and narrow set markers tested. Among one of the first use of DNA markers, restriction fragment length polymorphism (RFLP), randomly amplified polymorphic DNA (RAPD), and inter simple sequence repeat amplification (ISSR) markers were utilized to assess genome origins and genetic diversity among five species of the genus *Eleusine* (Salimath et al., 1995). RAPD markers have been the most widely used molecular markers to study genetic diversity of finger millet (Fakrudin et al., 2004; Babu et al., 2007; Kumari and Pande, 2010; Karad et al., 2013; Ramakrishnan et al., 2016). Diverse genotypes and varying number of RAPD markers have been employed to distinguish the finger millet accessions based on either their geographic origin (between Indian and African accessions and among Indian genotypes) or their pedigree, with the cultivated varieties shown to be genetically eroded due to continuous selection during breeding programs Additionally, other markers like RFLP and simple sequence repeat (SSR), Cytochrome P450-gene based markers have proved an effective marker system to establish genetic relationships among genotypes from various locations with respect to traits like plant architecture, yield, calcium and protein content (Dida et al., 2008; Panwar et al., 2010a,b; Kumar et al., 2012). Genic and genomic microsatellites are another excellent resource of molecular markers for finger millet. Unlike major cereals like rice and maize, only a few expressed sequence tags (ESTs) are available for finger millet in the public domains like NCBI. Despite this limitation, these ESTs have been extensively used to find the type, distribution, frequency of microsatellites and develop SSR primers (Arya et al., 2009; Naga et al., 2012; Babu et al., 2014b; Obidiegwu et al., 2014). In a cross-transferability study across a panel of nine cereals, including finger millet, 146 genic simple SSR primers were transferable among grasses with a conserved behavior within genotypes of finger millet (Yadav et al., 2014). Additionally, in the scarcity of genome sequence, comparative genomics allows us to project map-based orthologous candidate quantitative trait loci (QTLs) of interest which can be used for improvement of nutritional content. The availability of full sequence information of major cereal crops, like rice, maize and sorghum, it is now possible to do comparative genomic analysis for detecting alleles influencing several important traits in finger millet (Srinivasachary et al., 2007). This approach has been used to develop EST-SSRs from the opaque2 modifiers for grain protein and from the calcium transporters and calmodulin for calcium contents to analyze genetic diversity for these traits (Babu et al., 2014a; Nirgude et al., 2014). Babu et al. (2014a) performed association mapping for opaque2 modifiers (which influence the tryptophan content) and identified two QTLs for tryptophan and one QTL for protein content. Putative QTL for calcium content in finger millet grains using association mapping has been identified (Yadav et al., 2014; Kumar et al., 2015b). Thus, genetic diversity among finger millet genotypes has been efficiently differentiated based on their protein and calcium contents (Nirgude et al., 2014; Kumar et al., 2015b).

The identified molecular markers have promoted the potential to boost future breeding for nutritional improvement of finger millet especially in terms of mapping and tagging important QTLs and construction of genetic maps. The first genetic linkage map of finger millet, constructed at John Innes Centre, UK, covered 1,100 cM of genome across 27 linkage groups (LGs) using 212 RFLP markers (Gale and Devos, 1998). Since, 44% loci were detected by heterologous anchor probes, it can allow genome comparisons across the grasses and enrich genetic studies on finger millet (Gale and Devos, 1998). A more detailed map was generated by using several types of molecular markers like RFLP, AFLP, EST, and SSRs (Dida et al., 2007). The maps covered 18 major LGs of the finger millet genome, spanning 721 cM on the A genome and 787 cM on the B genome with seven or more markers at LOD 11. This map has helped to map important QTL that has polygenic control over several traits (Bharathi, 2011). However, for extensive use of this map, large-scale genome-wide marker information still needs to be generated.

### Gain-of-Function Approaches to Determine Gene Function in Finger Millet

An efficient regeneration system and methods of competent transformation form a prerequisite for crop improvement through genetic engineering. Several finger millet genotypes have been used for plant regeneration using different explants in tissue culture studies (Kothari et al., 2005; Reddy et al., 2008; Ceasar and Ignacimuthu, 2009). However, genetic engineering through transformation events is mostly limited to a few studies (**Table 3**). High leaf blast disease resistant finger millet transgenics have been successfully produced using biolistic or gene gun method (Gupta et al., 2001; Latha et al., 2005). Establishment of *Agrobacterium*-mediated genetic transformation has now paved the way for further studies in this millet and under optimal conditions up to 44.4% transformation efficiency has been obtained (Ceasar and Ignacimuthu, 2011; Sharma et al., 2011). Transgenic finger millet plants have been engineered toward higher biotic and abiotic stress tolerance (Ignacimuthu and Ceasar, 2012; Hema et al., 2014). An advanced and rapid method of microprotoplast-mediated chromosome transfer may allow transferring genes or chromosome from finger millet for different cell or species or genus-specific genetic manipulation (Yemets et al., 2013). Though, these entire developments offer a prolog for genetic engineering of stress resistance, focus also needs to be diverted toward nutritional quality improvement of finger millet in the future, most importantly after weighing all the associated risks and predicted benefits.

**Table 3:** Different transformation events in finger millet.

Method of transformation	Transformed explant	Promoter/reporter gene used/selectable marker	Target gene/biological process	Reference
Biolistic	Embryonic callus	CaMV35S/ActI/UqI/RbcS/Ft uidA	Efficiency of promoter in GUS reporter expression	Gupta et al., 2001
Biolistic	Embryonic callus	CaMV35S/uidA/bar	Antifungal protein (PIN) of prawn; transgenics resistant to leaf blast by *Pyricularia grisea*	Latha et al., 2005
Biolistic	Embryogenic callus	Ubiquitin or CaMV35S/β- and/or mutant α-tubulin genes		Yemets et al., 2013
*Agrobacterium*-mediated	Green nodular regenerative calli with meristematic nodules of seed origin	*Agrobacterium* strain EHA-105; CaMV35S/uidA/nptII	Establishment of transformation efficiency under different parameters	Sharma et al., 2011
*Agrobacterium*-mediated	Shoot apex	*Agrobacterium* strain LBA4404; CaMV35S/uidA/hptII	Establishment of transformation using shoot apex	Ceasar and Ignacimuthu, 2011
*Agrobacterium*-mediated	Callus	Maize ubiquitin promoter/uidA/hptII	Rice chitinase; transgenics resistant to leaf blast by *Pyricularia grisea*	Ignacimuthu and Ceasar, 2012
*Agrobacterium*-mediated	Embryogenic callus	CaMV35S/mtlD/uidA	Bacterial mannitol-1-phosphate dehydrogenase; transgenics exhibited multiple stress tolerance	Hema et al., 2014

### Transcriptomics

Gene expression profiling techniques or transcriptomics are the primary resource to identify important candidate genes involved in a biological process. A decade ago microarray and serial analyses of gene expression (SAGE) were the only approaches available for gene expression studies. The situation has changed dramatically during the last few years with the advent of next-generation sequencing and analytical tools (Wolf, 2013). Recently, a powerful alternative, called RNA-sequencing, has emerged for high throughput sequencing of cDNA (Soneson and Delorenzi, 2013). This is a powerful tool for studying nearly every gene regulating nutritionally important traits. For non-model organisms with limited genomic information, like finger millet, it can provides direct information about expression patterns of genes involved in nutrient accumulation and distribution during grain filling (Kumar et al., 2015a). Using metaanalysis of publically available gene expression data, a hypothetical model has been developed for elucidating the transport and distribution of calcium in embryonic seeds of cereals, specifically finger millet (Goel et al., 2012). Recently, large-scale transcriptome analysis has enabled identification of 82 unique calcium sensor genes from developing inflorescence from genotypes differing in their grain calcium content (Singh et al., 2014; Kumar et al., 2015a). These discovered candidate genes will provide a rich resource for further functional characterization. Gene expression of calmodulin and Cax1 transporter genes identified them to be highly expressed during the grain filling stages in high grain calcium genotype of finger millet (Nath et al., 2010; Kumar et al., 2014; Mirza et al., 2014). Similar global transcriptome analysis must be carried out to explore and understand possible mechanism for other biological traits for nutritional quality.

### Proteomics

Proteomics is the large-scale study of the complete set of proteins and analysis of their expression, structure and function (Kussmann et al., 2006). Proteome can be considered as a coupler between transcriptome of an organism and the ultimate responsive metabolome. The most widely used technique for proteomics is 2-D gel electrophoresis to separate the complex protein mixture. Mass spectrometry serves as a more specialized protein identification tool for different proteins (Fuchs et al., 2005; Harland, 2005; Kussmann et al., 2006; Mariman, 2006; Wang et al., 2006). Although, there are no reports of protein profiling for characterizing nutritive proteins, some studies identify proteins involved in calcium accumulation in finger millet. Elevated immunodetection of Calmodulin protein in the embryo and aleurone layer of high grain calcium finger millet genotype was correlated to stimulation of higher calcium accumulation during grain filling in developing seeds (Kumar et al., 2014). Another Calcium-binding protein, calreticulin, has been identified in developing spikes of finger millet using peptide mass fingerprinting (Singh et al., 2016). Identification of a finger millet peptide or protein of nutraceutical value through this approach will provide a cutting-edge dataset for use in research as well as therapy.

### Metabolomics and Ionomics

Metabolomics and ionomics are one of the recent “omics” technologies that allow the analysis of a low molecular weight biomolecules and elemental composition synthesized or consumed by a biological system, respectively. These technologies can provide a direct functional statement of a plant’s nutritive value by determination of its metabolic biochemistry, ionic contents, and phytochemicals (Sumner et al., 2003). Variation in nutritional content due to differential imbibition, transport and sequestration of minerals and ions depends not only on the organism’s physiological state but is also interrelated to its genetic makeup. Thus profiling for the complete metabolome can provide an effective phenotyping tool for the development of biomarkers for nutritional traits. Given that many of contributors toward finger millet’s nutritive value are either secondary metabolites like polyphenols or elements in their ionic form like Calcium, it warrants a metabolic profile evaluation to determine the best varieties for human consumption. Finger millet polyphenols have been identified through high performance liquid chromatography (HPLC), electrospray ionization mass spectrometry (ESI-MS), and nuclear magnetic resonance (NMR). These include derivatives of benzoic acid (gallic, protocatechuic, *p*-hydroxybenzoic, vanillic, and ferulic acids), cinnamic acid (syringic, *trans*-cinnamic, and *p*-coumaric acids); and quercetin (flavonoid; Chandrasekara and Shahidi, 2010; Banerjee et al., 2012). Subsequently, for biofortification of plants it is essential to expand this knowledge to other metabolites of nutritional importance.

### Bioinformatics and Systems Biology Approach

A multidisciplinary approach which aims to integrate biological sciences with computer studies and mathematics, called as systems biology, endeavors to quantitatively characterize the wealth of transcriptome, proteome, and metabalome datasets and simulate them to forecast an informational pathway (van Ommen and Stierum, 2002). In one of the preliminary studies, a mechanistic model in *Arabidopsis* was developed to identify and capture the key quantitative attributes of plant development and architecture (Mündermann et al., 2005). The systems-level approach has been used differentiate between C3 and C4 photosynthesis network in rice and maize, respectively, thereby providing a framework for economically valuable crops with improved carbon fixation (Wang et al., 2014). These works provide a working example to formulate a hypothesis that, in future systems biology approach can be extrapolated to explore nutraceutical properties of a crop.

## Conclusion and Future Prospects

The growing demand for healthier food, effectiveness and quality of consumed product and increased public and healthcare industry awareness are the major factors that have contributed toward the formation of a nutraceutical market that is envisioned to grow many folds in coming years. The Food and Drug Administration has also released regulations that support this emerging industry therefore fostering scientific research. Hence, an immediate goal should be identification of health benefitting factors to enhance the essential nutrient levels in staple crops to significantly impact human nutrition worldwide. Targeting of nutritionally important genes and proteins through the emerging biotechnology tools and techniques can lead to creation of ‘smart’ biofortified crops. Products from these value-added crops can help to cope with several problems such as protein-energy malnutrition. Research should establish impact of these products on the body’s absorption, defense, regulation of homeostasis and nervous systems, and then delve into hypo-allergenic foods and modern approaches to nutraceutical production. The initial research has shown finger millet to have a bright future in the nutraceutical industry and provides a scientific rationale for its use as an economically viable nutrient store to depreciate chronic pathologies. From the consumer’s perspective, establishment of finger millet as a nutraceutical can surpass the usual wait, efforts and cost inputs to bring conventional healthcare to the market and provide “self-care” for their satisfaction. Additionally, for a global scale, exploitation of its rich nutritional value assumes importance to provide food security, agricultural development, self-dependence and economic enhancement of developing countries. With the increasing knowledge about the nutraceutical properties of finger millet, the day is not far when this crop and its various products will find their place in the day to day menu of every individual.

## Author Contributions

AK conceptualized the manuscript. MM, SK, AKG, Sadhna Singh, MS wrote the manuscript. SG, BKB, Salej Sood assisted and edited the manuscript. SP and RSY contributed in critically revising the draft and updating the manuscript for publication.

## Conflict of Interest Statement

The authors declare that the research was conducted in the absence of any commercial or financial relationships that could be construed as a potential conflict of interest.

## Footnotes

1 http://www.fao.org/ag/agp/AGPC/doc/Gbase/data/Pf000235.HTM

2 http://exploreit.icrisat.org/page/small_millets/875

3 http://www.bioinnovate-africa.org/news/finger-millet-genomics-project-to-provide-researchers-with-better-tools-for-variety-production/

4 http://www.icrisat.org/crop-fingermillet.htm
